# Antibody Response to the BNT162b2 mRNA COVID-19 Vaccine in Subjects with Prior SARS-CoV-2 Infection

**DOI:** 10.3390/v13030422

**Published:** 2021-03-05

**Authors:** Federico Gobbi, Dora Buonfrate, Lucia Moro, Paola Rodari, Chiara Piubelli, Sara Caldrer, Silvia Riccetti, Alessandro Sinigaglia, Luisa Barzon

**Affiliations:** 1 Department of Infectious—Tropical Diseases and Microbiology, IRCCS Sacro Cuore Don Calabria Hospital, I-37024 Negrar, Italy; dora.buonfrate@sacrocuore.it (D.B.); lucia.moro@sacrocuore.it (L.M.); paola.rodari@sacrocuore.it (P.R.); chiara.piubelli@sacrocuore.it (C.P.); sara.caldrer@sacrocuore.it (S.C.); 2 Department of Molecular Medicine, University of Padova, I-35121 Padova, Italy; silvia.riccetti@unipd.it (S.R.); alessandro.sinigaglia@unipd.it (A.S.); 3 Microbiology and Virology Unit, Padova University Hospital, I-35128 Padova, Italy

**Keywords:** COVID-19 vaccine, neutralizing antibody, anti-spike RBD IgG antibody, BNT162b2 vaccine, SARS-CoV-2, immune response, vaccination, immunogenicity, reactogenicity, vaccine doses

## Abstract

Although antibody levels progressively decrease following SARS-CoV-2 infection, the immune memory persists for months. Thus, individuals who naturally contracted SARS-CoV-2 are expected to develop a more rapid and sustained response to COVID-19 vaccines than naïve individuals. In this study, we analyzed the dynamics of the antibody response to the BNT162b2 mRNA COVID-19 vaccine in six healthcare workers who contracted SARS-CoV-2 in March 2020, in comparison to nine control subjects without a previous infection. The vaccine was well tolerated by both groups, with no significant difference in the frequency of vaccine-associated side effects, with the exception of local pain, which was more common in previously infected subjects. Overall, the titers of neutralizing antibodies were markedly higher in response to the vaccine than after natural infection. In all subjects with pre-existing immunity, a rapid increase in anti-spike receptor-binding domain (RBD) IgG antibodies and neutralizing antibody titers was observed one week after the first dose, which seemed to act as a booster. Notably, in previously infected individuals, neutralizing antibody titers 7 days after the first vaccine dose were not significantly different from those observed in naïve subjects 7 days after the second vaccine dose. These results suggest that, in previously infected people, a single dose of the vaccine might be sufficient to induce an effective response.

## 1. Introduction

As of 1 February 2021, more than 99 million cases of coronavirus disease 2019 (COVID-19) have been confirmed, and about two million deaths have been reported to the World Health Organization (WHO) [1]. Globally, joint efforts to gain control of the severe acute respiratory syndrome coronavirus 2 (SARS-CoV-2) pandemic resulted in the unprecedented and rapid development of vaccines, with the COVID-19 mRNA vaccines developed by Pfizer/BioNTech and Moderna being the first to receive emergency use authorization from the Food and Drug Administration (FDA) and the European Medicines Agency (EMA).

In Italy, the vaccination campaign started in the final days of December 2020, with the first available doses of the BNT162b2 mRNA COVID-19 vaccine (Pfizer/BioNTech) being delivered to healthcare workers and elderly residents in nursing homes. The vaccine is currently offered on a voluntary basis to all individuals, irrespective of a prior SARS-CoV-2 infection, due to equivocal data on the duration of naturally acquired immunity and documented (though still limited) cases of re-infection [2,3,4].

Nevertheless, although a decline in antibody levels (including neutralizing antibodies) is observed over time in infected individuals, the immune memory, which consists of memory B cells, antibodies, memory CD4+ T cells, and/or memory CD8+ T cells, persists for months and even increases with time in the case of memory B cells against the SARS-CoV-2 spike protein [5,6]. Thus, we might expect that subjects with a previous natural SARS-CoV-2 infection might develop a more rapid and sustained response to a COVID-19 vaccine than individuals who were not infected. Understanding immune memory of SARS-CoV-2 and its implications for the protective immunity induced by COVID-19 vaccination is crucial for the optimization of COVID-19 vaccine immunization programs.

In this study, we report preliminary data on the dynamics of the antibody response against SARS-CoV-2, pre- and post-vaccination with the BNT162b2 mRNA COVID-19 vaccine, observed in six health workers who were infected with SARS-CoV-2 in March 2020, in comparison with the antibody response to vaccination in naïve subjects.

## 2. Materials and Methods

### 2.1. Ethics

This work is part of a larger study that received clearance from the local Ethics Committee (Comitato Etico per la Sperimentazione Clinica delle Province di Verona e Rovigo) on 8 April 2020 (study protocol n. 2624CESC).

### 2.2. Participants and Setting

Since March 2020, at the Istituto di Ricovero e Cura a Carattere Scientifico (IRCCS) Sacro Cuore Don Calabria Hospital, Negrar, Verona, Italy, the staff involved in patient care and laboratory procedures have undergone molecular testing for SARS-CoV-2 infection in the case of suspected symptoms. Moreover, periodic screening with molecular testing has been offered to all staff, irrespective of symptoms, since April 2020.

At the same Institution, the vaccination campaign with the BNT162b2 mRNA COVID-19 vaccine (Pfizer, NY, USA, and BioNTech, Mainz, Germany) began on 1 January 2021. For the purpose of this study, consent regarding the donation of serum samples pre- and post-COVID-19 vaccination, aimed at evaluating antibody dynamics following vaccination, was requested from all hospital staff. In this study, we included six healthcare workers involved in patient care who were infected during the first wave of SARS-CoV-2 in spring 2020 (Group 1) and a control group (Group 2) of nine healthcare workers who did not contract SARS-CoV-2, who were randomly selected from the same department as those who did contract the virus. Blood samples were drawn from study subjects on the day that they were inoculated with the first vaccine dose (Day 0), at the time of the second vaccine dose (Day 21) and 7 days after the first and the second doses of the vaccine. Among the Group 1 subjects, four were followed up serologically for the purpose of the study at 2, 4, 5 and 7 months after symptom onset. In these subjects, the pre-vaccination serum sample corresponded to 9 months after symptom onset.

### 2.3. Laboratory Tests

In Group 1 subjects, a diagnosis of SARS-CoV-2 infection was confirmed by molecular testing of nasopharyngeal swabs. Briefly, nasopharyngeal swabs were processed according to the routinely used diagnostic protocol, standardized following the WHO guidelines [7]. RNA extraction was performed by an automated method (MagNApure LC RNA isolation kit-High performance, Roche), according to the manufacturer’s instructions. Real-time reverse transcription polymerase chain reaction (qRT-PCR) tests were executed using the US Centers for Disease Control and Prevention (CDC) 2019-nCoV qRT-PCR Diagnostic Panel assay and protocol [8] with a limit of detection (LOD) of 10 copies/well. As an internal control, the human beta actin *ACTB* target was used.

IgG/IgM antibodies to SARS-CoV-2 were detected using the following serological tests:

- An automated chemiluminescent microparticle immunoassay (CMIA) test for the qualitative detection of IgM antibodies to the spike protein of SARS-CoV-2 (SARS-CoV-2 IgM, Abbott Laboratories, IL, USA). According to the manufacturer’s instructions, serum samples were considered positive when the output index was ≥1.0 AU/mL.
- An automated CMIA test for the qualitative detection of IgG antibodies to the nucleocapsid protein of SARS-CoV-2 (SARS-CoV-2 IgG, Abbott Laboratories). Serum samples were considered positive when the output index was ≥1.4 AU/mL.
- An automated CMIA test for the qualitative and quantitative detection of IgG antibodies to the spike receptor-binding domain (RBD) of SARS-CoV-2 (SARS-CoV-2 IgG II Quant assay, Abbott Laboratories). The LOD of the assay was 6.9 AU/mL; the upper limit of quantification was 80,000 AU/mL.

The three assays were run on an ARCHITECT i System (Abbott), according to the manufacturer’s instructions.

Neutralizing antibodies against SARS-CoV-2 were titrated by an in-house SARS-CoV-2 microneutralization assay performed at the Department of Molecular Medicine, University of Padua. Briefly, serum samples were diluted in Eagle’s minimal essential medium, starting from 1:10 in a twofold dilution series, mixed 1:1 with a virus solution containing 100 fifty-percent tissue culture infectious dose (TCID50) of SARS-CoV-2 isolated from a symptomatic patient. Fifty microliters of the virus–serum mixtures were incubated for 1 h at 37 °C, 5% CO_2_, followed by inoculation into tissue culture microtiter plates containing Vero E6 cells grown as monolayers at 37 °C, 5% CO_2_. At 72 h of incubation, wells were observed via light microscopy for the presence of cytopathic effects compared to the virus control and were stained with Gram’s crystal violet solution, plus 5% formaldehyde, for 30 min. All neutralization tests were performed in duplicate. Neutralizing antibody titers were defined as the reciprocal of the highest dilution of the serum that showed 50% neutralization of cytopathic effects in a microneutralization assay. Neutralizing antibody titers ≥20 were considered positive.

### 2.4. Statistical Analysis

Data were represented as the median value and interquartile range (IQR) and as the geometric mean titer (GMT) ± 95% confidence interval (95% CI). Comparisons between groups were conducted using Mann–Whitney U-tests with continuity correction. Statistical significance was defined as *p* < 0.05. All statistical analyses were performed using Statistica™ software, version 13.4.0.14 (TIBCO Software Inc., Palo Alto, CA, USA).

## 3. Results

### 3.1. Case Description

All six healthcare workers included in Group 1 had a mild disease (Table 1) and were quarantined at home until they recovered from their symptoms and had two consecutively negative SARS-CoV-2 molecular tests on their nasopharyngeal swabs, performed at an interval of at least 24 h, according to the national guidelines. The cases included three female and three male individuals, with a median age of 44.5 years (IQR 40.5–47.0 years). Symptom onset was reported from 17 March 2020 to 22 March 2020. The first positive SARS-CoV-2 qRT-PCR result was obtained 1–4 days after symptom onset and the median duration of quarantine was 30 days, with a range of 24–41 days.

Control healthcare workers without SARS-CoV-2 infection (Group 2) included four females and five males, with a median age of 36 years (IQR, 33–57 years).

### 3.2. Reactogenicity of BNT162b2 mRNA COVID-19 Vaccine

Vaccination was well tolerated by both Group 1 and Group 2 subjects. The most common vaccine-associated side effects were local pain and asthenia (Table 2). Side effects occurred with comparable frequency in Group 1 and Group 2 subjects, with the exception of local pain, which was reported more frequently by Group 1. In both groups, side effects were more frequent after the second vaccine dose (Table 2).

### 3.3. Antibody Responses after Natural SARS-CoV-2 Infection and Vaccination with BNT162b2 mRNA Vaccine

**Figure 1:**
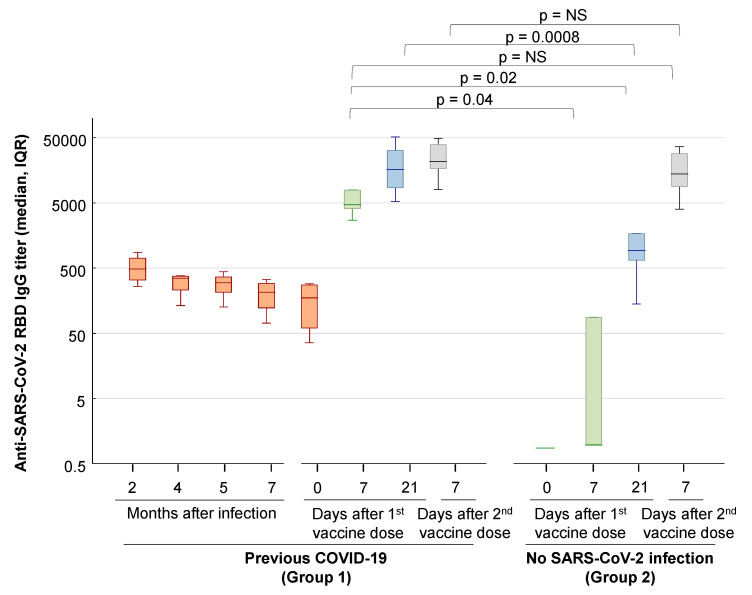
Anti-SARS-CoV-2 IgG antibody responses after natural SARS-CoV-2 infection and after vaccination with the BNT162b2 mRNA COVID-19 vaccine. Titers of IgG anti-SARS-CoV-2 spike receptor-binding domain (RBD) were measured with a chemiluminescent microparticle immunoassay (SARS-CoV-2 IgG II Quant assay, Abbott). The results are reported in the box–whisker plots as median IgG AU/mL, 25% to 75% percentile, and non-outlier range. Blood samples were drawn from Group 1 (*n* = 6 subjects with prior SARS-CoV-2 infection) and Group 2 (*n* = 9 subjects without prior SARS-CoV-2 infection) on days 0, 7 and 21 after the first vaccine dose and on day 7 after the second vaccine dose. The second vaccine dose was administered on day 21. In four out of the six subjects in Group 1, serology testing was also performed at 2, 4, 5, and 7 months after natural SARS-CoV-2 infection. The *p* values of Mann–Whitney U-tests with continuity correction are reported. NS: *p* value not statistically significant.

**Figure 2:**
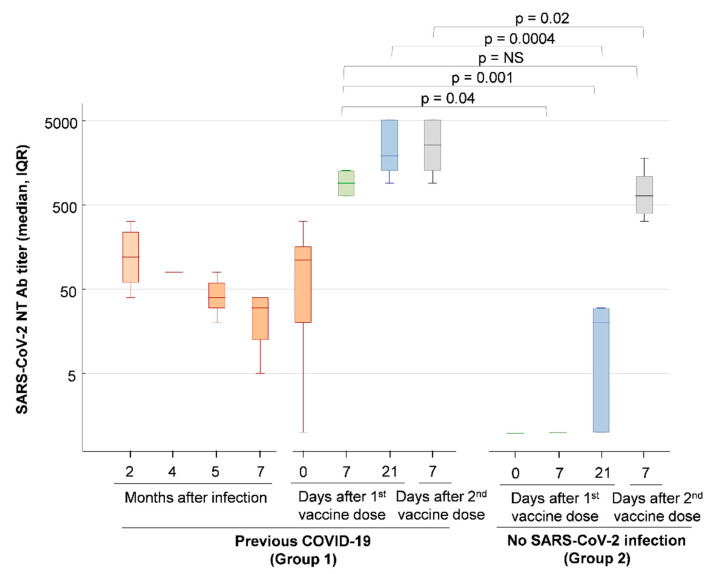
Anti-SARS-CoV-2 neutralizing antibody responses after natural SARS-CoV-2 infection and after vaccination with the BNT162b2 mRNA COVID-19 vaccine. SARS-CoV-2 neutralizing antibody titers were measured with a microneutralization assay using live virus. The results are reported in the box–whisker plots as median neutralization (NT) titers, 25% to 75% percentile, and non-outlier range. Blood samples were drawn from Group 1 (*n* = 6 subjects with prior SARS-CoV-2 infection) and Group 2 (*n* = 9 subjects without prior SARS-CoV-2 infection) on days 0, 7 and 21 after the first vaccine dose and on day 7 after the second vaccine dose. The second vaccine dose was administered on day 21. In four out of the six subjects in Group 1, serology testing was also performed at 2, 4, 5, and 7 months after natural SARS-CoV-2 infection. The *p* values of Mann–Whitney U-tests with continuity correction are reported. NS: *p* value not statistically significant.

Four out of the six cases in Group 1 (with SARS-CoV-2 infection) were followed-up with antibody testing at 2, 4, 5 and 7 months after symptom onset. Overall, anti-SARS-CoV-2 spike RBD IgG titers and neutralizing antibody titers progressively declined during follow-up (Figure 1 and Figure 2).

Following the first dose of the BNT162b2 mRNA COVID-19 vaccine, all Group 1 subjects rapidly developed high titer anti-SARS-CoV-2 RBD IgG antibodies and neutralizing antibodies, which were about 10–100-fold higher than pre-vaccination titers, as shown by the results of serology testing 7 days after the first vaccine dose (Figure 1 and Figure 2). In contrast, Group 2 subjects, in whom prior SARS-CoV-2 infection was excluded by screenings with molecular and serology tests, had slower antibody responses and reached high titer antibodies only after the second vaccine dose (Figure 1 and Figure 2). In particular, half of the Group 2 subjects had neutralizing antibody titers <20 on the day of administration of the second vaccine dose (Day 21), but all mounted anti-SARS-CoV-2 neutralizing antibodies after the second vaccine dose, with titers >320 in all subjects on day 7 after the second dose. Notably, the titers of SARS-CoV-2-neutralizing antibodies measured in Group 1 subjects 7 days after the first vaccine dose were not significantly different from the neutralizing antibody titers measured 7 days after the second vaccine dose in naïve Group 2 subjects (GMT, 95% CI: 906, 552–1348 vs. 670, 364–1228, *p* = NS).

**Figure 3:**
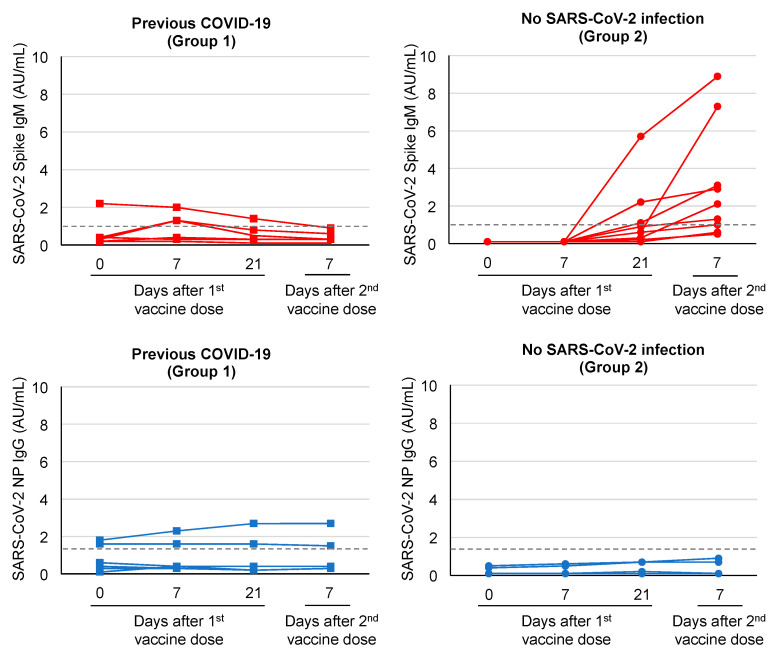
Anti-SARS-CoV-2 Spike IgM antibody (upper panels) and anti-SARS-CoV-2 nucleocapsid protein (NP) IgG antibody (lower panels) levels in healthcare workers with previous COVID-19 infection (Group 1, *n* = 6) and in healthcare works without prior SARS-CoV-2 infection (Group 2, *n* = 9). Blood samples were drawn on days 0, 7 and 21 after the first BNT162b2 mRNA vaccine dose and on day 7 after the second vaccine dose. The second BNT162b2 mRNA vaccine dose was administered on day 21. Antibodies were determined by qualitative chemiluminescent microparticle immunoassay (CMIA) assays (Abbott Laboratories) and reported as AU/mL values. Dashed horizontal lines represent cutoff values.

The analyses of IgM antibodies against the spike protein of SARS-CoV-2 showed transiently positive results only in two subjects from Group 1, on day 7 after the first vaccine dose, in agreement with prior immunization results. In contrast, most Group 2 subjects mounted anti-spike IgM antibodies, which were detectable in four out of nine subjects on day 21 after the first vaccine dose and in seven out of nine subjects on day 7 after the second vaccine dose (Figure 3).

As expected, IgG antibodies against the nucleocapsid protein of SARS-CoV-2 were detectable in all Group 1 subjects after SARS-CoV-2 infection and their levels (output index) showed a decline during follow-up (at 2 and 7 months after infection, median 4.6, range 3.2–5.7 AU/mL, and median 1.2, range 0.2–2.0 AU/mL, respectively). At the time of the first vaccine dose, i.e., at 9 months after infection, only two out of six Group 1 subjects still had detectable anti-nucleocapsid protein IgG antibodies, whose levels remained stable after the first and second doses of the COVID-19 vaccine (Figure 3). In contrast, at the time of the first vaccine dose, all Group 2 subjects had no detectable anti-nucleocapsid protein IgG antibodies and remained negative following vaccination, confirming that they had not been infected with SARS-CoV-2 (Figure 3).

## 4. Discussion

In this study, we report the dynamics of antibodies against SARS-CoV-2, including neutralizing antibodies, in a group of healthcare workers who contracted SARS-CoV-2 in March 2020 and received two doses of the BNT162b2 mRNA COVID-19 vaccine in January 2021. The reactogenicity and antibody response to vaccination was compared with that of a control group of healthcare workers from the same department, who were not previously infected with SARS-CoV-2.

The vaccine was well tolerated both in previously infected subjects and in naïve individuals, with no significant difference in the frequency of vaccine-associated side effects, with the exception of local pain, which was more common in previously infected subjects. In contrast with our findings, a study by Krammer et al. [9], with a larger number of subjects, reported more severe reactogenicity to mRNA vaccines in individuals with pre-existing immunity, since they developed systemic symptoms more frequently than individuals who were seronegative at vaccination, while local side effects occurred with comparable frequency in the two groups.

In our study, subjects with prior SARS-CoV-2 infection showed a decline in antibody titers with time, which rapidly and markedly increased after COVID-19 vaccination, as demonstrated by the 10–100-fold increase in anti-spike RBD IgG and neutralizing antibody titers in serum samples collected one week after the first vaccine dose. At the time of administration of the second dose of the vaccine, that is, about 3 weeks after the first dose, all subjects with prior SARS-CoV-2 infection showed a further increase in anti-SARS-CoV-2 spike RBD IgG titers and neutralizing antibody titers, at levels similar to those observed after the second vaccine dose, suggesting that a plateau was reached.

In contrast, only half of the naïve controls had developed neutralizing antibodies at the time of the second vaccine dose, while all control individuals developed high neutralizing antibody titers one week after the second dose of the vaccine. These results are in agreement with a recent study, which showed that a single BNT162b2 vaccine dose induced low-level neutralizing antibodies and T-cell responses in naïve individuals [10].

Overall, the levels of neutralizing antibodies were markedly higher in response to the vaccine than after natural infection. In all subjects with pre-existing immunity, a rapid increase in neutralizing antibody titers was observed one week after the first dose, which seemed to act as a booster. Indeed, such a rapid response was not demonstrated in controls. Notably, in previously infected individuals, SARS-CoV-2 neutralizing antibody titers measured in serum samples collected 7 days after the first dose of the BNT162b2 mRNA COVID-19 vaccine were not significantly different from the neutralizing antibody titers observed in naïve subjects only 7 days after the second vaccine dose. This might suggest that, in previously infected people, even those who had a mild disease, such as the subjects of this study, a single dose of the vaccine might be sufficient to induce an effective response.

This aspect is of paramount importance when considering how we can conserve doses of the vaccine, e.g., in the context of scarce availability. In agreement with our observations, recent studies on both the Pfizer/BioNTech and Moderna mRNA COVID-19 vaccines reported significantly higher antibody titers in vaccinees with a previous infection than in naïve vaccinees [9,10,11,12,13,14,15,16]. In particular, analyses of the antibody response to the first BNT162b2 dose in previously infected individuals demonstrated an increase in anti-spike IgG and neutralizing antibody titers of more than 140 times that of peak pre-vaccine levels, and at levels significantly higher than those found in infection-naïve individuals [11]. Likewise, a single BNT162b2 vaccine dose induces significantly higher virus neutralization titers and stronger T-cell responses to the SARS-CoV-2 spike protein in previously infected individuals than in naïve individuals [10]. In addition, an investigation into the immunogenicity of the BNT162b2 mRNA vaccine in a large cohort of Israeli healthcare workers showed that, 21 days after the first vaccine dose, those with a prior infection had antibody titers one order of magnitude higher than naïve individuals, regardless of the presence of detectable IgG antibodies pre-vaccination [17]. Although our study was performed with a small group of subjects, it nevertheless provides important information about the dynamics of the antibody response to vaccination. In fact, in contrast with the above reports, our study includes a homogeneous group of individuals who were infected 9 months before vaccination and who had low anti-SARS-CoV-2 RBD IgG antibodies and neutralizing antibody titers at the time of vaccination. The rapid and robust neutralizing antibody response to vaccination in these subjects confirms the persistence of the immunological memory of SARS-CoV-2 [5]. In addition, our study showed that the second BNT162b2 vaccine dose did not result in a further increase in anti-RBD IgG and virus-neutralizing titers in subjects with a previous SARS-CoV-2 infection.

One key consideration is whether vaccinees should be tested for IgG antibodies before vaccination. In our study, anti-SARS-CoV-2 RBD IgG antibodies detected using CMIA Quant showed dynamics comparable to neutralizing antibodies and, in some cases, the test was more sensitive, since anti-RBD IgG antibodies could be detected earlier than neutralizing antibodies. The availability of standardized automated immunoassays, such as the CMIA Quant test used in this study, for the detection of antibodies targeting SARS-CoV-2 spike proteins (or its RBD as a proxy for neutralizing antibodies) [18,19] will make it easier to determine antibody responses to SARS-CoV-2 vaccines in most laboratories, while neutralizing assays can only be performed in a few laboratories.

As expected, IgG antibodies against the nucleocapsid protein detected using a CMIA did not show an increase in response to the vaccine (the cases with positive IgG antibodies were still harboring them in response to the natural infection). This test could be used to retrospectively identify individuals who naturally contracted SARS-CoV-2 in order to distinguish them from vaccinated individuals. However, in our study, the sensitivity of the anti-SARS-CoV-2 nucleocapsid protein IgG CMIA was low, since only two out of six subjects had detectable IgG antibodies 9 months after infection. Likewise, testing IgM antibodies against nucleocapsid protein could be useful when attempting to identify a recent infection, as anti-spike protein IgM antibodies are also induced by vaccination. However, anti-spike protein IgM antibodies showed a different path when compared between infected healthcare workers and controls, with most of the latter testing positive after the second dose of the vaccine, while only two individuals with pre-existing immunity had a slight, transient increase in IgM antibodies one week after the first vaccine dose, in agreement with secondary immunization. Since it was not possible to discern a clear correlation between vaccination and IgM antibodies detected by CMIA, this test might not be suitable for evaluating the response to vaccination.

The main limitation of this study is the small number of individuals included. However, we believe that these preliminary data are useful and that they raise important questions that should be investigated further in larger cohorts of COVID-19 vaccinees.

## 5. Conclusions

SARS-CoV-2 neutralizing antibodies showed a rapid, marked increase after the first dose of the BNT162b2 mRNA COVID-19 vaccine in a group of previously infected individuals and reached titers similar to those observed in naïve subjects only after the second vaccine dose. These findings raise the question of whether a single vaccine shot might be sufficient in the presence of a previous infection. CMIA Quant demonstrated its usefulness in evaluating the response to the vaccine in individuals with and without previous infection. Further studies on larger cohorts of patients are required to confirm these findings, which are relevant to the conservation of vaccine supplies and to the implementation of strategies to monitor the response to vaccination across a larger number of laboratories.

## Figures and Tables

**Table 1:** Demographic and clinical findings in six healthcare workers with SARS-CoV-2 infection (Group 1).

ID	Age Range, Sex	Symptoms	Quarantine Duration (Days)
1	30s, female	Fever (max 38.5 °C) for 4 days, asthenia, mild cough, diarrhea (1 day), ageusia, anosmia	25
2	40s, male	Fever (max 38 °C) for 1 day, asthenia, arthralgias, anosmia	24
3	40s, male	Asthenia, oral ulcers, ageusia, anosmia	29
4	40s, female	Fever (max 38 °C) for 3 days, asthenia, ageusia	41
5	30s, female	Fever (max 38.5 °C), asthenia, ageusia, anosmia	32
6	40s, male	Asthenia, anosmia	35

**Table 2:** Vaccine-associated side effects experienced after the first and the second dose of BNT162b2 mRNA COVID-19 vaccine in Group 1 (with previous SARS-CoV-2 infection) and in Group 2 (naïve) subjects.

Vaccine-Associated Side Effects	Group 1 (*n =* 6)		Group 2 (*n =* 9)	
	First Dose	Second Dose	First Dose	Second Dose
Local pain	6 (100%)	5 (83%)	3 (33%)	4 (44%)
Asthenia	0	3 (50%)	2 (22%)	4 (44%)
Fever > 37.5 °C	0	1 (17%)	0	2 (22%)
Headache	0	0	1 (11%)	3 (33%)
Myalgia	0	1 (17%)	0	2 (22%)
Adenopathy	1 (17%)	0	0	0
Lymphangitis	0	0	0	1 (11%)
Nausea	0	0	0	1 (11%)

## Data Availability

The data presented in this study are openly available in the Open Science Framework (OSF) repository at https://osf.io/d6kq8/ accessed on 16 February 2021.

## References

[1] World Health Organization (WHO) Coronavirus Disease (COVID-19) Weekly Epidemiological Update and Weekly Operational Update. https://covid19.who.int/.

[2] Marot S., Malet I., Leducq V., Zafilaza K., Sterlin D., Planas D., Gothland A., Jary A., Dorgham K., Bruel T. (2021). Rapid decline of neutralizing antibodies against SARS-CoV-2 among infected healthcare workers. Nat. Commun..

[3] Dimeglio C., Herin F., Miedougé M., Martin-Blondel G., Soulat J.M., Izopet J. (2021). Protection of healthcare workers against SARS-CoV-2 reinfection. Clin. Infect. Dis..

[4] Hall V., Foulkes S., Charlett A., Atti A., Monk E.J.M., Simmons R., Wellington E., Cole M.J., Saei A., Oguti B. (2020). Do antibody positive healthcare workers have lower SARS-CoV-2 infection rates than antibody negative healthcare workers? Large multi-centre prospective cohort study (the SIREN study), England: June to November. medRxiv.

[5] Dan J.M., Mateus J., Kato Y., Hastie K.M., Yu E.D., Faliti C.E., Grifoni A., Ramirez S.I., Haupt S., Frazier A. (2021). Immunological memory to SARS-CoV-2 assessed for up to 8 months after infection. Science.

[6] Gaebler C., Wang Z., Lorenzi J.C.C., Muecksch F., Finkin S., Tokuyama M., Cho A., Jankovic M., Schaefer-Babajew D., Oliveira T.Y. (2021). Evolution of antibody immunity to SARS-CoV-2. Nature.

[7] World Health Organization Diagnostic Testing for SARS-CoV-2. https://www.who.int/publications/i/item/diagnostic-testing-for-sars-cov-2.

[8] Centers for Disease Control and Prevention COVID-19. Research Use Only 2019-Novel Coronavirus (2019-nCoV) Real-Time RT-PCR Primers and Probes. https://www.cdc.gov/coronavirus/2019-ncov/lab/rt-pcr-panel-primer-probes.html.

[9] Krammer F., Srivastava K., Simon V., the PARIS team (2021). Robust spike antibody responses and increased reactogenicity in seropositive individuals after a single dose of SARS-CoV-2 mRNA vaccine. medRxiv.

[10] Prendecki M., Clarke C., Brown J., Cox A., Gleeson S., Guckian M., Randell P., Dalla Pria A., Lightstone L., Xu X.-N. (2021). Effect of previous SARS-CoV-2 infection on humoral and T-cell responses to single-dose BNT162b2 vaccine. Lancet.

[11] Manisty C., Otter A.D., Treibel T.A., McKnight Á., Altmann D.M., Brooks T., Noursadeghi M., Boyton R.J., Semper A., Moon J.C. (2021). Antibody response to first BNT162b2 dose in previously SARS-CoV-2-infected individuals. Lancet.

[12] Saadat S., Rikhtegaran Tehrani Z., Logue J., Newman M., Frieman M.B., Harris A.D., Sajadi M.M. (2021). Single dose vaccination in healthcare workers previously infected with SARS-CoV-2. medRxiv.

[13] Stamatatos L., Czartoski J., Wan Y.-H., Homad L.J., Rubin V., Glantz H., Neradilek M., Seydoux E., Jennewein M.F., MacCamy A.J. (2021). Antibodies elicited by SARS-CoV-2 infection and boosted by vaccination neutralize an emerging variant and SARS-CoV-1. medRxiv.

[14] Levi R., Azzolini E., Pozzi C., Ubaldi L., Lagioia M., Mantovani A., Rescigno M. (2021). A cautionary note on recall vaccination in ex-COVID-19 subjects. medRxiv.

[15] Tada T., Dcosta B.M., Samanovic-Golden M., Herati R.S., Cornelius A., Mulligan M.J., Landau N.R. (2021). Neutralization of viruses with European, South African, and United States SARS-CoV-2 variant spike proteins by convalescent sera and BNT162b2 mRNA vaccine-elicited antibodies. bioRxiv.

[16] Bradley T., Grundberg E., Selvarangan R. (2021). Antibody responses boosted in seropositive healthcare workers after single dose of SARS-CoV-2 mRNA vaccine. medRxiv.

[17] Abu Jabal K., Ben-Amram H., Beiruti K., Batheesh Y., Sussan C., Zarka S., Edelstein M. (2021). Impact of age, ethnicity, sex and prior infection status on immunogenicity following a single dose of the BNT162b2 mRNA COVID-19 vaccine: Real-world evidence from healthcare workers, Israel, December 2020 to January 2021. Euro Surveill..

[18] Bonelli F., Sarasini A., Zierold C., Calleri M., Bonetti A., Vismara C., Blocki F.A., Pallavicini L., Chinali A., Campisi D. (2020). Clinical and analytical performance of an automated serological test that identifies S1/S2-neutralizing IgG in COVID-19 patients semiquantitatively. J. Clin. Microbiol..

[19] Meyer B., Reimerink J., Torriani G., Brouwer F., Godeke G.J., Yerly S., Hoogerwerf M., Vuilleumier N., Kaiser L., Eckerle I. (2020). Validation and clinical evaluation of a SARS-CoV-2 surrogate virus neutralisation test (sVNT). Emerg. Microbes Infect..

